# Effect of Juglone and Other Allelochemicals in Walnut Leaves on Yield, Quality and Metabolites of Snack Cucumber (*Cucumis sativus* L.)

**DOI:** 10.3390/foods12020371

**Published:** 2023-01-12

**Authors:** Aljaz Medic, Tilen Zamljen, Ana Slatnar, Metka Hudina, Mariana Cecilia Grohar, Robert Veberic

**Affiliations:** Department of Agronomy, Biotechnical Faculty, University of Ljubljana, SI 1000 Ljubljana, Slovenia

**Keywords:** allelopathy, cucumber, naphthoquinones, juglone (5-hydroxy-1,4-naphthalenedione), phenolic compounds, toxic residue, HPLC, mass spectrometry, LC-MS, snack cucumber

## Abstract

The consumption of fresh vegetables has been consistently associated with numerous health benefits. However, several factors (such as allelochemicals) influence yield, quality, and metabolites, which inevitably affect the fruit quality and health benefits. The present study was conducted to investigate the yield, quality, metabolic responses, and potential toxicity of *Cucumis sativus* grown in juglone-containing soils. For the treatments, pure juglone (100 µM, 1 mM) and walnut leaf extracts (100 µM) in soil concentrations found in walnut orchards were used. A total of 36 phenolic compounds were identified and quantified in fruits, leaves, and roots using a mass spectrometer coupled with high-performance liquid chromatography. We concluded that juglone at a concentration of 100 µM or walnut leaf extract at the same juglone concentration does not affect the yield of *C. sativus*, while juglone at a concentration of 1 mM strongly affects it. In the case of juglone, juglone itself was found only in the roots of *C. sativus*, but not in the leaves or fruits, so *C. sativus* fruits are considered safe for cultivation in juglone-containing soils. However, this could prove problematic if the plants grown are tubers or root vegetables. The data suggest that juglone itself inhibits secondary metabolism in the plant, making it more susceptible to stress and pathogen attacks.

## 1. Introduction

*Cucumis sativus* L. (cucumber) is a member of the Cucurbitaceae family, which comprises 90 genera and 750 species. *C. sativus* is cultivated in nearly all countries of temperate zones [1]. It is one of the oldest cultivated vegetable crops and the second most cultivated Cucurbitaceae in the world after watermelon [2]. *C. sativus* is thermophilic, growing best at temperatures above 20 °C. Consumer and processing industry demand dictates the external appearance such as the size, shape, and color of the fruit, as well as the internal quality (taste, health benefits) [1].

The consumption of fresh vegetables is an essential part of the human diet and has been consistently associated with numerous health-promoting benefits (reduced obesity, fewer diet-related diseases, anti-inflammatory effects, etc.) [3]. In addition to vitamins, phytochemicals are considered the most important factors contributing to the health benefits and nutritional value of vegetables. Phytochemicals are classified into six major classes (phenols, alkaloids, organosulfur compounds, phytosterols, carotenoids, and nitrogen-containing compounds) [4]. Of these, phenols (phenolic compounds) along with carotenoids are the most represented and best studied group of phytochemicals. They are associated with many health benefits (e.g., anti-inflammatory, antimicrobial, antiallergenic, and antioxidant effects) and are also touted as potentially protective against some generative diseases (e.g., cancer, diabetes, and cardiovascular disease) [5,6,7]. Phenols also play an important role in the quality of vegetables by affecting their appearance, flavor, and stability [8].

From a plant perspective, phenols play an important role in the defense against pathogens, predators, and abiotic and biotic stresses. When a plant is exposed to stressful conditions, the phenolic content increases and helps the plant overcome the stress. However, due to higher phenolic content synthesis, the plant consumes more energy and nutrients that were actually intended for its growth and primary functions [9,10]. Since a higher phenolic content is associated with higher vegetable quality, plant stress is considered beneficial to some extent. However, higher phenolic content is the result of plant stress, which has a negative impact on plant growth and thus yield, so a balance must be struck between quality and yield. While some stress factors can be controlled by agronomic practices (e.g., pest and disease control, fertilization, and irrigation), others are uncontrollable or difficult to control (e.g., fungi and plant residues in the soil) [11,12].

For example, plant residues release allelochemicals into the soil. Some of these allelochemicals have positive effects on plants (e.g., stimulating their growth), while others have negative effects (e.g., inhibiting their growth). Depending on the susceptibility of the plants, these allelochemicals can cause chlorosis, wilting, and malformation, thus reducing plant vigor, hindering plant growth and development, slowing or preventing germination, and increasing plant susceptibility to disease, which can eventually lead to plant collapse [13]. In horticulture, this is particularly problematic when one crop follows another.

This problem occurs especially when walnut (*Juglans regia* L.) orchards are replaced by other crops, since walnuts contain one of the first and best studied allelochemicals-juglone or 5-hydroxy-1,4-naphthalenedione [12]. It is believed that the hydrojuglone glycosides (nontoxic forms of juglone) are washed from the leaves by rain or released from walnut residues. In the soil, they are then oxidized to the toxic form of juglone, which, after uptake by the roots of surrounding plants, impairs their growth and development [14]. The juglone content in soil under walnut trees has been reported to range from 0.25 mM to 3.25 mM, depending on the season [15]. Juglone is thought to penetrate the plasma membrane of cells and induce depolarization by blocking K^+^ channels [16]. As a result, juglone inhibits shoot and especially root growth [12] and nutrient uptake [14]. In addition, juglone exhibits antimicrobial and antifungal activity and attacks naturally occurring symbionts, destroying a useful source of nutrients [14].

Although it is common practice to clear an old orchard to make way for new crops, very little is known about the long- and short-term effects of allelochemicals still actively released from plant residues or still present in the soil and how these allelochemicals might affect the quality, toxicity, and yield of future crops. Most studies addressing juglone have examined seed germination [12,17,18], rather than the yield or quality of mature plants, where only one study was found [19], while no study has examined the whole plant molecular response to juglone and walnut leaf extract. In addition, the available studies have generally focused on the effects of juglone itself, and few studies have looked at the effects of walnut leaf extracts [12,19] which could better answer the question of whether juglone is indeed the only allelochemical in walnut leaf extracts.

In the absence of studies, the natural objective of the present study was to investigate the molecular response of the whole plant (leaves, roots, and fruits) and fruit quality and yield to known concentrations of juglone alone and in walnut leaf extract with concentrations of juglone normally found in the soil of walnut orchards. *C. sativus* was used for this purpose because it produces fruit at short intervals and was therefore best for the sampling purpose, especially to study the time required for juglone to act on the plant and its fruit.

## 2. Materials and Methods

### 2.1. Plant Material

Snack-type cucumbers were used for this experiment because they have smaller leaves and fruits and ripen faster than conventional slicing cucumbers (2–3 days after fruit set), being only about 10 cm long when picked. Therefore, we were able to shorten the picking interval, as we observed in previous experiments [19] that the effect of juglone on the plants was already visible after a few days. Juglone concentrations were based on our previous studies [12,19]. Plants were grown on a hydroponic system and treated similarly to Medic et al. [19] with two control treatments of (i) the water control (denoted as K1 in the text) and (ii) as the juglone extraction medium and vehicle control (0.17% dimethyl sulfoxide (DMSO)), 0.17% ethanol in H_2_O (referred to as K2 in the text), positive control treatments with pure juglone prepared for final juglone concentrations of (iii) 1 mM (referred to as 10-3 in the text) and (iv) 100 µM in the extraction medium (referred to as 10-4 in the text), and (v) leaf juglone extract prepared for the final juglone concentration of 100 µM in the extraction medium (referred to as LEAF 10-4 in the text). As explained earlier, juglone was dissolved in the extraction medium because juglone is only partially soluble in water (52 mg/L). Therefore, the required concentration of 1 mM control juglone in water alone could not be achieved.

### 2.2. Growing Conditions

For the experiment, a nutrient film technique (NFT) hydroponic system was used. The experiment took place in the greenhouse (the experimental field of the Department of Agronomy in the Biotechnical Faculty, of the University of Ljubljana, Slovenia) to better control the environmental factors that could affect the results, while the NFT system was used to exclude the influence of soil as a medium and to control the juglone concentrations in the treatments. Five NFT systems were used, one for each treatment, with each NFT system comprising only one row to ensure that no shading by other plants affected the experiment. Each row consisted of 10 plants, resulting in a total of 5 biological replicates per measure (5 for yield and visual determination and 5 for metabolomics studies).

Plants were germinated from seeds in a greenhouse, and sown in pots filled with standard peat substrate and after the appearance of the third leaf, the substrate was washed from the roots of the seedlings. Then, the seedlings were placed in plastic pots filled with rockwool and left for 3 weeks for the plants to root into the rockwool substrate before being placed on an NFT system. There, they were grown for 4 weeks with the addition of nutrients as previously reported [20]. After the plants were acclimatized for 4 weeks, the treatments were added. The plants of snack-type cucumbers (*C. sativus* ‘Hopeline F1’) were grown from seeds obtained from Austrosaat AG, Wien, Austria.

### 2.3. Chemicals, Plant Material, and Preparation of J. regia Leaf Extract

The walnut leaves used for the preparation of the walnut leaf extract were obtained from the Experimental Field for Nut Crops in Maribor (Slovenia).

The control juglone and leaf extract dilutions were prepared to the protocol as previously described by Medic et al. [12].

The following standards were used for the quantification of phenolic compounds: p-coumaric acid (Fluka Chemie GmbH, Buchs, Switzerland) for *p*-coumaric acid; caffeic acid (Sigma-Aldrich Chemie GmbH, Steinheim, Germany) for caffeic acid derivatives; ferulic acid (Fluka Chemie GmbH) for ferulic acid hexoside derivatives and feruloyl hexoside; gallic acid (Sigma-Aldrich Chemie GmbH) for hydrogallic acid and benzoic acid; sinapic acid (Sigma-Aldrich Chemie GmbH) for sinapic acid hexoside derivatives; quercetin-3-*O*-rutinoside (Sigma-Aldrich Chemie GmbH) for quercetin-3-*O*-rutinoside; quercetin-3-*O*-rhamnoside (Sigma-Aldrich Chemie GmbH) for quercetin-3-*O*-rhamnoside; isorhamnetin-3-*O*-glucoside (Extrasynthese, Genay, France) for isorhamnetin-3-*O*-glucoside; juglone (Sigma-Aldrich Chemie GmbH) for juglone; apigenin-7-*O*-glucoside (Sigma-Aldrich Chemie GmbH) for isovitexin derivatives; and luteolin-7-*O*-glucoside (Sigma-Aldrich Chemie GmbH) for isoscoparin derivatives.

A Milli-Q water purification system by Millipore (Bedford, MA, USA) was used to bidistillate and purify the water used to prepare the samples. The acetonitrile and formic acid used as mobile phases for MS analysis were of HPLC-MS grade from Fluka Chemie GmbH. The methanol used for the extraction of the phenolic compounds was of HPLC-MS grade from Sigma-Aldrich Chemie GmbH.

### 2.4. Sampling of the Plants

The cucumber fruits were collected prior to the treatments being added (day 0), after 3 days, and after 6 days of the added being added. Fully developed leaves (20–40 cm from the base of the plant) were collected on day 0 and on day 6, and the roots were collected on day 6. Day 6 was the last day of sampling because the juglone-treated plants did not bear fruit after that and began to collapse. Day 0 was set when all the plants acclimatized and one week after the last plant started bearing fruit (to have a better uniformity). Looking at fruit sampling, there were five biological replicates (two to four fruits per plant depending on the fruit set). The fruit from five plants were used for yield measurements and for measurements of DA index (index ΔA), Brix, firmness, and color. Fruits from the other five plants were picked, placed in paper bags, frozen using liquid nitrogen, and transported to the laboratory (Department of Agronomy, Biotechnical Faculty, University of Ljubljana, Slovenia). For metabolomics, ample leaves and roots were sampled in five replicates (two plants per replicate). The leaf and root samples were then lyophilized, then all samples were ground to a powder using the IKA^®^ A11 basic analytical mill (IKA^®^-Werke GmbH & Co., KG, Staufen, Germany) and stored at −20 °C until further analysis.

A T.R. Turoni (Forlì, Italy) digital penetrometer with an 4.8 mm blunt piston, expressed in Newton [N], was used to measure fruit firmness as previously reported by Suojala-Ahlfors [21]. A 53500 DA METER^®^ (T.R. Turoni Srl, Forlì, Italy) was used to measure the index ΔA, which produces an index of the state of maturation of the fruit based on the difference of absorbance at two wavelengths. A digital refractometer (Milwaukee Digital Brix Refractometer MA871, Rocky Mount, NC, USA) was used for measuring the soluble solid content, and a CR-300 Chroma colorimeter (Minolta Co., Osaka, Japan) was used to measure fruit skin color parameters. Color parameters were as follows: L* (lightness), where values vary from 0 (black) to 100 (white); a* (positive values represent red and negative green); b* (positive values represent yellow and negative values represent blue); C* (colorfulness), where higher values represent a more intense color; and *h* (hue angle), which is expressed in degrees.

### 2.5. Extraction of the Phenolic Compounds

The extraction protocol was as previously described in detail by Medic et al. [12,19]. Briefly, 1 g of fresh fruit samples or 100 mg of previously lyophilized samples of leaves and roots were extracted at a tissue-to-solution ratio of 1:3 for fruit samples and 1:30 for leaves and roots (*w*/*v*). The extraction medium used was 80% methanol and 3% formic acid in water.

### 2.6. HPLC–Mass Spectrometry Analysis of Individual Phenolic Compounds

A Vanquish UHPLC system (Thermo Scientific, Waltham, MA, USA) was used for the analysis of individual phenolic compounds. The diode detector was set at 350 nm for flavonols and flavones and at 280 nm for the other phenolic compounds. The recorded spectra ranged from 200 nm to 600 nm. For the separation of the compounds, a Gemini 150 mm × 4.60 mm; 3 µm (Phenomenex, Torrance, CA, USA) C18 column was used. The column used for the separation of phenolic compounds was operated at 25 °C. Solvents, elution flow rate, gradient, washing, and reconditioning of the column between the samples were performed as described by Medic et al. [19].

An LTQ XL tandem mass spectrometer (Thermo Scientific, Waltham, MA, USA) coupled with a Vanquish UHPLC system with heated electrospray ionization was used for the identification of phenolic compounds. It was operated in the negative ion mode. Parameters were as described by Medic et al. [19] and scans were performed from *m*/*z* 50 to 2000. For data acquisition, the Xcalibur 2.2 software (Thermo Fischer Scientific Institute, Waltham, MA, USA) was used.

An HPLC-MS was also used to quantify juglone in the treatments and to test whether juglone residues remained in the plants themselves, which could be considered toxic to consumers.

For the known compounds, external standards were used for identification and quantification, while literature data and MS fragmentation were used for the identification of the unknown compounds. A standard similar to the identified compound was used for its quantification. Individual phenolic compounds, total flavones, total flavonols, total hydroxybenzoic acids, total hydroxycinnamic acids, total analyzed phenolics content (TAPC), and total analyzed phenolics content without juglone content (TAPCWJ) are all expressed as mg/kg fresh weight for fruits and mg/kg dry weight for roots and leaves.

### 2.7. Statistical Analysis

The data were collated using R commander (package Rcmdr) version 2.7.1. (Team, R.D.C., 2008, Stanford, CA, USA) and Microsoft Excel (MS Office 2016). Five biological repetitions (for each methodology) were performed. Data are presented as means ± standard errors (SE). To determine the differences between treatments, a one-way analysis of variance (ANOVA) with Tukey tests was used. Statistical means were calculated at a 95% confidence level to determine the significance of the differences.

## 3. Results and Discussion

### 3.1. Identification of Individual Phenolic Compounds

A total of 36 compounds were identified based on standard fragmentation and the previous literature (pseudomolecular ions (i.e., [M-H]^−^) and specific fragmentation patterns (i.e., MS^2^, MS^3^)). A total of 13 phenolic compounds were identified in the fruit, 29 in the leaves, and 5 in the roots of *C. sativus*. Many of these compounds were identified for the first time in the fruit and especially the leaves of *C. sativus*, whereas all phenolic compounds were identified for the first time in the roots of *C. sativus* because to the best of our knowledge there are no previous studies on the phenolic compounds in the roots of *C. sativus*. The identified phenolic compounds as well as their retention times, the pseudomolecular ions identified in the negative ion mode, the fragment ions, and the relative abundance of the fragment ions are listed in Table 1. Chromatograms of the different tissues studied can also be found in the Supplementary Material (Figures S1–S3).

A total of 24 flavones were identified; of those, there were mainly isovitexin and isoscoparin glycosides and derivatives. Isovitexin derivatives and glycosides were identified by their specific fragmentation patterns of MS^n^ ions *m*/*z* 593, 413, and 293, and isoscoparin derivatives and glycosides were identified by their specific fragmentation patterns of MS^n^ ions *m*/*z* 443, 413, and 323, as previously reported in cucumber fruit and fruit peel extracts by Abou-Zaid et al. [22] and Mukherjee et al. [23]. Most of these phenolic compounds have been reported for the first time in leaves of *C. sativus*.

Of the three flavonols quercetin-3-*O*-rutinoside, quercetin-3-*O*-rhamnoside, and isorhamnetin-3-*O*-glucoside and one naphthoquinone (juglone), all were identified using external standards and their fragmentations.

The remaining phenolic compounds were identified based on external standards and their fragmentation pattern as well as previous reports by Ezzat et al. [24], Abu-Reidah et al. [25], and Ul Haq et al. [26].

### 3.2. Effects of Juglone Treatments on Cucumber Yield and Quality

A look at Table 2 shows that the highest weight of fruit was obtained under walnut leaf extract treatment, which is in agreement with our previous results indicating that walnut leaf extracts contain, in addition to juglone, other nutrients and beneficial allelochemicals that can stimulate plant growth and increase fruit weight at lower concentrations but inhibit them at higher concentrations [12,19]. From the concentration of 100 µM juglone in walnut leaf extract, we can conclude that this concentration has a positive effect on the fruit weight of *C. sativus*. There was no difference between the control treatments (K1 and K2) and 10-4, suggesting that juglone at a concentration of 10^−4^ M (100 µM) has no effect on fruit weight, in contrast to a juglone concentration of 10^−3^ M (1 mM), which greatly reduces the fruit weight almost tenfold compared to the control treatments.

Looking at some of the fruit quality parameters, we find that there is no difference between treatments when looking at the ΔA, except for 10-3, which indicates that the fruits of the 10-3 treatment are riper than the fruits of the other treatments. Similar is the case for fruit firmness and soluble solids content, where the highest value was observed in the 10-3 treatment. This indicates that even if the fruits are 10 times lighter, they tend to ripen faster. This could be the result of a lower water content in the fruits due to the collapse of the roots and their ability to absorb water and nutrients normally through the tissues, as previously reported [19], which is probably one of the effects of juglone [16]. Interestingly, both the treatment with pure juglone (10-4) and the treatment with walnut leaf extract, which contained the same amount of juglone, did not differ in terms of the soluble solids content and were both higher than the control treatments, suggesting that lower concentrations of juglone may stimulate primary metabolism in plants, as previously reported for biostimulants [27]. Regarding the color parameters, it is noted that there is no color difference between the controls and the lower concentrations of pure juglone or walnut leaf extracts, while the fruits of the juglone 10-3 treatment were brighter and more yellow compared to the other treatments, which is also one of the symptoms of the juglone effect described previously [14]. Overall, it can be concluded that fruits grown in soils with juglone concentrations higher than 1 mM would be severely damaged and unmarketable, both in terms of a lower yield (tenfold lower fruit weight) and quality, producing more yellow fruits with non-specific characteristics (such as sweeter or harder). Interestingly, lower concentrations of juglone in the soil (100 µM) have no effect on other parameters of the fruits, except for the soluble solids content, making the fruits slightly sweeter compared to those grown in soils without juglone, which could possibly affect the acceptance of the fruits by consumers.

### 3.3. Effects of Juglone Treatments on Phenolic Composition of Cucumber Fruit

**Figure 1:**
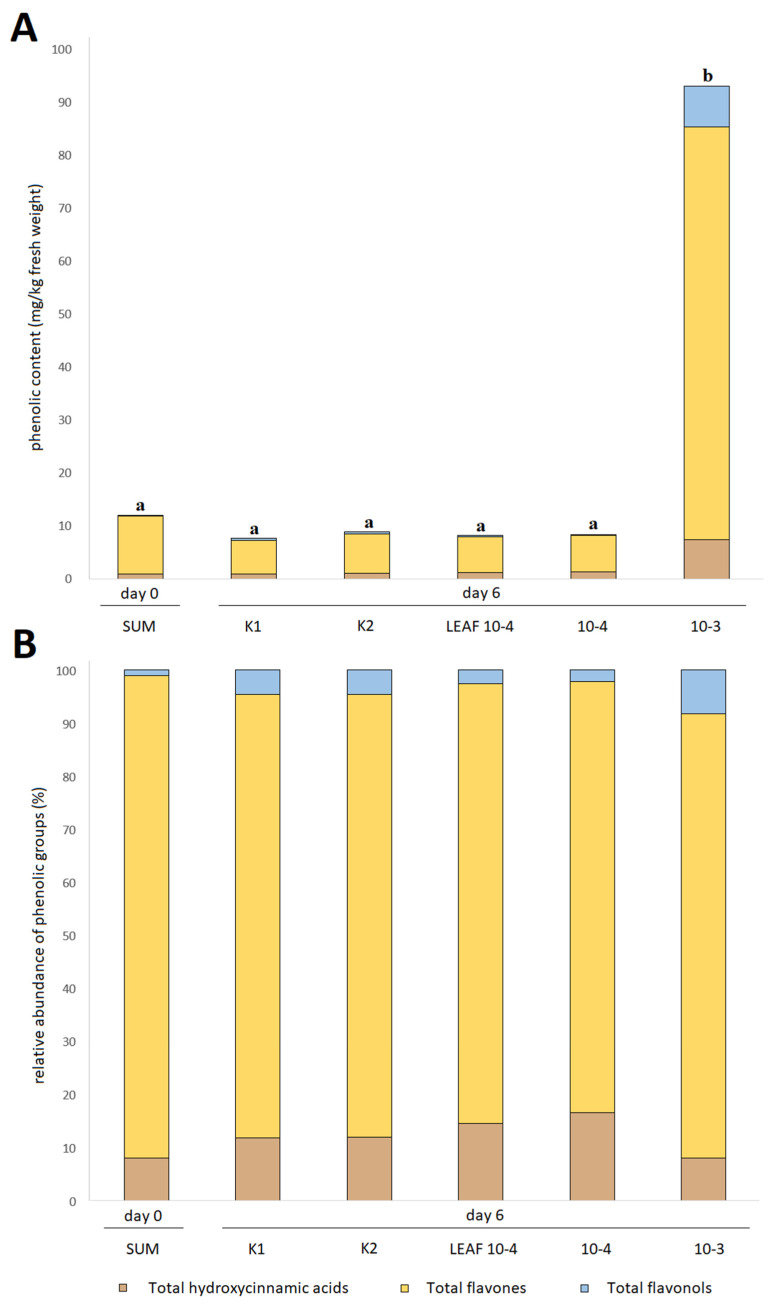
Contents of the total phenolic groups identified in fruit of *C. sativus* expressed relative to fresh weight (**A**) and as proportions of the total phenolic groups identified (**B**). SUM, fruit collected prior to adding any treatments from all plants; K1, water control; K2, juglone extraction medium and vehicle control (0.17% dimethyl sulfoxide (DMSO)), 0.17% ethanol in H_2_O; 10-4 positive control treatment with pure juglone prepared for final juglone concentration of 100 µM in the extraction medium; 10-3 positive control treatment with pure juglone prepared for final juglone concentration of 1 mM; LEAF 10-4 leaf juglone extract prepared for final juglone concentration of 100 µM in the extraction medium; different letters across the treatments represent significant difference (*p* < 0.05) between the total analyzed phenolics content (TAPC).

Similarly to biostimulants, allelochemicals also alter the phenolic profile and stimulate the secondary metabolism of plants, affecting their flavor, internal quality, and shelf life [19]. Some allelochemicals can even be absorbed by plants and thus become toxic to consumers [28]. In the case of juglone, juglone itself was found only in the roots of *C. sativus*, but not in the leaves or fruits, which is why the fruits of *C. sativus* are considered safe for cultivation in juglone-containing soils. This is likely due to the fact that juglone does not dissolve well in polar solvents and is therefore difficult to transport through the plant [16], but this could prove problematic if the plants grown are tubers or root vegetables that are in direct contact with juglone. Figure 1 shows that the highest total analyzed phenolic content (TAPC) was found in the juglone 10-3 treatment, where the content was up to 10 times higher than in the fruits of the other treatments. Interestingly, the fruit weight of the fruit treated with the 10-3 treatment was up to ten times lower, so the TAPC content per fruit was similar to the fruit of the other treatments, but it was more concentrated because the fruit were lighter. This is to be expected to some extent because plants in stress situations shift their focus from growth and cell growth to secondary metabolism to protect the plant from stress or pathogens, which requires a lot of energy and nutrients for its synthesis [9,29].

Looking at the relative content of phenolic groups, we find that there is not much difference in the composition of phenols between treatments, a similar situation as with the yield and quality parameters described earlier. However, we can note that the highest content of flavonols and flavones was reported for the 10-3 treatment, which is expected since the increased synthesis of flavones and especially flavonols has been previously correlated with induced stress [9] and they are considered one of the most important phenolic groups in plant defense against stress and pathogens [29].

Both flavones and especially flavonols are also usually associated with health-promoting effects as they are among the most beneficial phenolic groups for human health [30]. As shown in Table 3, no changes in the metabolism of phenolic compounds were observed after 3 days of application of the treatments, while changes in the secondary metabolism of the fruit indicated that the plant became under stress between 3 and 6 days after the application of the treatment, indicating that the fruit is not affected immediately after the application of juglone, but only after several days.

### 3.4. Effects of Juglone Treatments on Phenolic Composition of Cucumber Leaves

**Figure 2:**
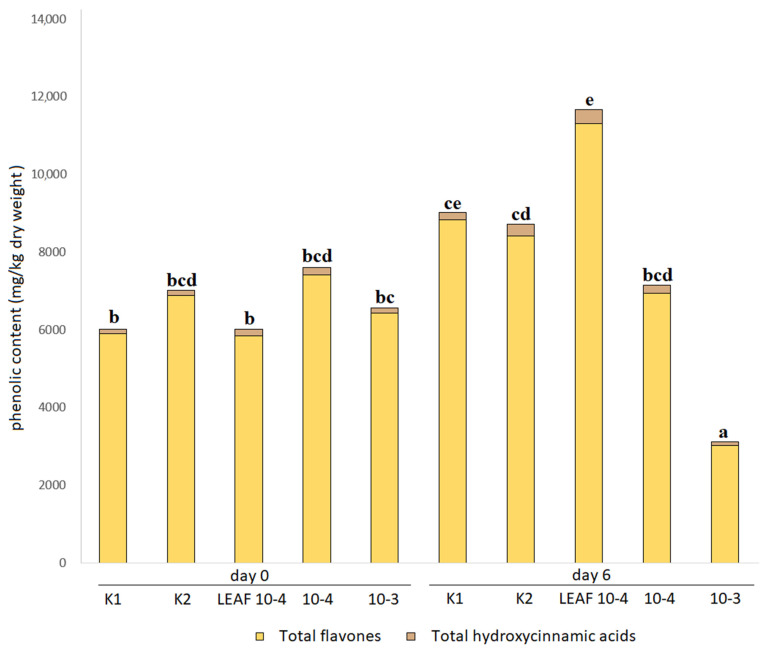
Contents of the total phenolic groups identified in the leaves of *C. sativus* expressed relative to dry weight. K1, water control; K2, juglone extraction medium and vehicle control (0.17% dimethyl sulfoxide (DMSO)), 0.17% ethanol in H_2_O; 10-4 positive control treatment with pure juglone prepared for final juglone concentration of 100 µM in the extraction medium; 10-3 positive control treatment with pure juglone prepared for final juglone concentration of 1 mM; LEAF 10-4 leaf juglone extract prepared for final juglone concentration of 100 µM in the extraction medium; different letters across the treatments represent significant difference (*p* < 0.05) between the TAPC.

Although the focus of this study was to investigate the effect of juglone on the fruit (the edible part) of *C. sativus*, the effect of juglone on other plant tissues (leaves and roots) was also examined, primarily to better understand the translocation of juglone through different plant tissues. A look at the effects of different treatments of juglone and walnut leaf extract on the secondary metabolism of *C. sativus* in Figure 2 shows that the highest TAPC was reported for the treatment LEAF 10-4, which is consistent with our previous results [12,19] and indicates that walnut leaf extract, similar to biostimulants, also alters the phenolic profile and stimulates plant secondary metabolism, making the plant more robust and tolerant to stress conditions and pathogens. Interestingly, the TAPC content in leaves was up to three times lower in the 10-3 treatment than in the other treatments, in contrast to TAPC in fruits. This and the data in Table 4 suggest that the effect of juglone on leaves occurs much more rapidly than the previously observed effect on fruit, where the effects of juglone treatments were observed after 6 days, whereas here they occurred on the third day of measurement. Interestingly, the treatment with pure juglone (10-4) had a different effect on leaf metabolites than the treatment with walnut leaf extract (LEAF 10-4), which stimulated phenol production. The data suggest that juglone itself inhibits secondary metabolism in the plant, making it more susceptible to stress and pathogenic attack. This is likely due to the fact that juglone destroys the roots and thus the plant’s ability to absorb water and nutrients normally through the tissues, and thus the plant’s ability to successfully produce these valuable defense compounds. Ultimately, this leads to the collapse of the plant, as has been reported in other studies [12,14,19,31].

### 3.5. Effects of Juglone Treatments on Phenolic Composition of Cucumber Roots

**Figure 3:**
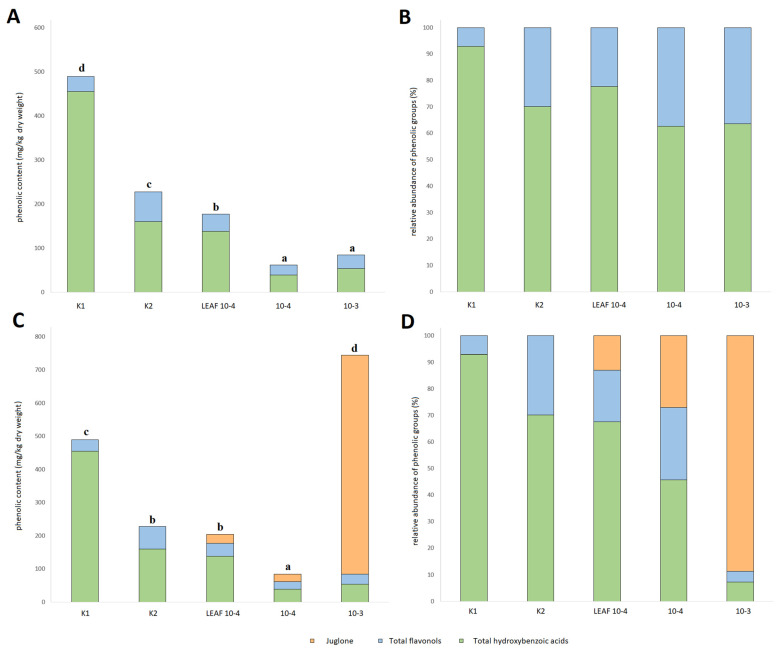
Content of total phenolic groups identified in the roots of *C. sativus* expressed relative to dry weight (**A**) and as a proportion of total phenolic groups identified (**B**) without considering juglone content and expressed relative to dry weight (**C**) and as a proportion of total phenolic groups identified (**D**) with considering juglone content. K1, water control; K2, juglone extraction medium and vehicle control (0.17% dimethyl sulfoxide (DMSO)), 0.17% ethanol in H_2_O; 10-4 positive control treatment with pure juglone prepared for final juglone concentration of 100 µM in the extraction medium; 10-3 positive control treatment with pure juglone prepared for final juglone concentration of 1 mM; LEAF 10-4 leaf juglone extract prepared for final juglone concentration of 100 µM in the extraction medium; different letters across the treatments represent significant difference (*p* < 0.05) between the TAPC.

In contrast to the metabolic profile of the fruits and leaves of *C. sativus*, the roots contained juglone in both pure juglone and walnut leaf extract treatments (Figure 3 and Table 5). There were no differences in the juglone content in walnut leaf extract and pure juglone, confirming that the juglone content in walnut leaf extract was correctly quantified before treatment. Interestingly, the highest TAPC content was reported for K1 and the second highest for K2. This could be due to the fact that hydroxybenzoic acids were the main phenolic compounds identified in the roots and their role was not associated with plant defense mechanisms. When the content of the other phenolic groups identified was considered, there were no differences between treatments except for K2. Similar to the leaves, the roots treated with juglone and walnut leaf extract had the lowest content, which could be due to the fact that the juglone destroys the roots themselves. In addition, considering the juglone content, the highest TAPC with juglone or TAPCWJ was obviously reported for the 10-3 treatment. Overall, no clear conclusions could be drawn from the metabolism of the roots.

## 4. Conclusions

We can conclude that juglone at a concentration of 100 µM or walnut leaf extract at the same concentration of juglone does not affect the yield of *C. sativus*, while juglone at a concentration of 1 mM affects it severely. From the concentration of 100 µM juglone in walnut leaf extract, it can be concluded that this concentration still has a positive effect on the yield of *C. sativus*. In the case of juglone, juglone itself was found only in the roots of *C. sativus*, but not in the leaves or fruits, which is why *C. sativus* fruits are considered safe for cultivation in juglone-containing soils. This is likely due to the fact that juglone does not dissolve well in polar solvents and is therefore difficult to transport through the plant. However, this could prove problematic if the plants grown are tubers or root vegetables in direct contact with juglone. The effect of juglone on leaves occurs much faster than the previously observed effect on fruits, where the effects of juglone treatments were observed after six days, while here they appeared on the third day of measurement. The data suggest that juglone itself inhibits secondary metabolism in the plant, making it more susceptible to stress and pathogenic attack. This is probably due to the fact that juglone destroys the roots and thus the ability of the plants to absorb water and nutrients normally through the tissues, and thus the ability of the plants to successfully produce these valuable defense substances.

## Figures and Tables

**Table 1:** Tentative identification of the 36 phenolic compounds from fruit, leaves, and roots of *C. sativus*.

Compound	Rt (min)	[M-H]^−^ (*m*/*z*)	MS^2^ (*m*/*z*)	MS^3^ (*m*/*z*)	Fruit	Leaves	Roots
Caffeic acid derivative 1	10.21	447	401(100), 179(4)	179(100)		x	
Meloside A (Isovitexin-2-*O*-glucoside) 1	10.81	755	593(100), 455(71), 413(45), 293(12)	473(100), 413(93), 293(20)		x	
Caffeic acid derivative 2	11.50	447	401(100), 179(4)	179(100)		x	
Ferulic acid hexoside derivative	12.12	401	355(100), 193(20)	193(100)	x	x	
Meloside A (Isovitexin-2-*O*-glucoside) 2	12.75	755	593(100), 455(71), 413(45), 293(12)	473(100), 413(93), 293(20)	x	x	
Isoscoparin-2-*O*-(6-(E)-feruoyl)-glucoside	13.65	785	623(100), 485(85), 443(42), 323(9)	503(100), 443(69), 323(13)		x	
Sinapic acid hexoside derivative	14.34	431	385(100)	205(100), 153(74), 223(54), 161(23)	x	x	
Feruoyl hexoside	14.43	355	193(100)		x		
Saponarin (isovitexin-7-*O*-glucoside)-4-*O*-glucoside	14.49	623	503(100), 533(24), 341(3)		x	x	
Isoscoparin-2-*O*-(6-(E)-feruoyl)-dihexoside 1	14.83	961	799(100), 443(15), 323(5)	443(100), 323(32), 413(26)		x	
Isovitexin-2-*O*-(6-(E)-feruoyl)-dihexoside 1	14.98	931	769(100), 413(7), 293(2)	413(100), 593(70), 293(47)	x	x	
Isovitexin-2-*O*-(6-(E)-*p*-coumaroyl)-dihexoside 1	15.22	901	413(100), 739(98), 781(46), 293(40), 323(20), 341(19)	293(100)	x	x	
Isoscoparin-2-*O*-(6-(E)-feruoyl)-dihexoside 2	15.78	961	799(100), 443(13), 323(4)	443(100), 323(32), 413(26)		x	
Isovitexin-2-*O*-(6-(E)-feruoyl)-dihexoside 2	16.06	931	769(100), 413(7), 293(2)	413(100), 593(70), 293(47)	x	x	
Isovitexin-2-*O*-(6-(E)-*p*-coumaroyl)-dihexoside 2	16.15	901	413(100), 739(84), 781(45), 293(34), 341(22), 323(17)	293(100)	x	x	
Isoscoparin-2-*O*-(6-(E)-feruoyl)-dihexoside 3	16.60	961	799(100), 443(13), 323(3)	443(100), 323(32), 413(26)		x	
Isovitexin-2-*O*-(6-(E)-feruoyl)-dihexoside 3	16.71	931	769(100), 413(7), 293(3)	413(100), 593(70), 293(47)		x	
Isovitexin-2-*O*-(6-(E)-*p*-coumaroyl)-dihexoside 3	16.96	901	413(100), 739(92), 781(47), 293(37), 323(18), 341(17)	293(100)		x	
Benzoic acid	17.02	121	93(100), 92(17), 121(16), 75(8), 77(5)				x
Isoscoparin-2-*O*-(6-(E)-feruoyl)-rhamnoside-glucoside	17.43	947	785(100), 429(5)			x	
Meloside A (Isovitexin-2-*O*-glucoside)-glucoside	17.58	917	755(100)			x	
Meloside A (Isovitexin-2-*O*-glucoside)-glucoside derivative	17.84	1073	755(100), 911(98), 893(17), 867(15)			x	
Isovitexin-8-C-galactoside	18.18	593	413(100), 293(22)	293(100)		x	
Vicenin 2 (Isovitexin-8-C-glucoside)	19.04	593	413(100), 293(21)	293(100)	x	x	
Isoscoparin-2-*O*-glucoside	19.54	623	443(100), 323(21)	323(100), 365(4)		x	
*p*-Coumaric acid	20.41	163	119(100)			x	
Quercetin-3-*O*-rutinoside	20.43	609	301(100), 300(25), 179(3)				x
Isovitexin-2-*O*-(6-(E)-feruoyl)-hexoside derivative	21.10	897	853(100), 593(30), 413(5), 293(2)	593(100), 413(17), 293(4)		x	
Isoscoparin-2-*O*-(6-(E)-feruoyl)-derivative	21.41	799	443(100), 623(68), 323(31), 413(18), 593(15)	323(100)		x	
Isoscoparin-2-*O*-(6-(E)-feruoyl)-rhamnoside	21.68	769	623(100), 443(70), 413(11), 323(21)	443(100), 323(22)	x	x	
Isovitexin-2-*O*-(6-(E)-feruoyl)-glucoside	21.83	769	413(100), 293(46), 593(65)	293(100), 335(14)		x	
Isovitexin-2-*O*-(6-(E)-*p*-coumaroyl)-glucoside	21.99	739	593(100), 413(70), 293(26), 619(10)	413(100), 293(27)	x	x	
Quercetin-3-*O*-rhamnoside	23.15	447	301(100), 300(20), 179(2)				x
Isorhamnetin-3-*O*-glucoside	23.45	477	314(100), 315(38), 357(29)		x		
Hydrogalic acid	23.47	187	125(100)				x
Juglone (5-hydroxy-1,4-naphthalenedione)	26.02	189	161(100)				x

Rt, retention time; x, presence of the compound identified; [M-H]^−^, pseudo-molecular ion identified in negative ion mode; (), relative abundance of fragment ions; MS^2^, fragment ions obtained from pseudomolecular ion in negative ion mode; MS^3^, fragment ions obtained from the most abundant pseudomolecular ion of MS^2^ fragmentation.

**Table 2:** *C. sativus* yield measurements and fruit measurements of DA index (ΔA), soluble solids content, firmness, and color parameters, between different treatments on day 6.

Measurement	K1	K2	LEAF 10-4	10-4	10-3
Average weight (g)	39.5 ± 5.6 c	36.5 ± 5.4 bc	49.1 ± 5.3 d	35.2 ± 4.9 bc	4.3 ± 1.0 a
ΔA	2.2 ± 0.0 a	2.1 ± 0.0 a	2.1 ± 0.0 a	2.1 ± 0.0 a	2.3 ± 0.1 b
Soluble solids content (°Bx)	3.4 ± 0.2 a	3.5 ± 0.1 a	4.2 ± 0.1 b	4.7 ± 0.1 b	6.2 ± 0.5 c
Firmness (N)	1.8 ± 0.3 a	1.6 ± 0.2 a	1.5 ± 0.3 a	1.5 ± 0.2 a	4.6 ± 0.6 b
Color parameters					
L*	31.7 ± 1.1 a	33.5 ± 0.5 ab	31.8 ± 0.4 a	31.6 ± 0.8 a	35.9 ± 0.8 b
a*	−4.6 ± 0.2 a	−5.4 ± 0.1 a	−4.7 ± 0.1 a	−4.4 ± 0.2 a	−3.9 ± 0.2 b
b*	17.1 ± 1.3 a	20.5 ± 0.7 ab	18.2 ± 0.5 ab	17.1 ± 0.9 a	21.7 ± 0.8 b
C*	17.7 ± 1.4 a	21.1 ± 0.7 ab	18.9 ± 0.5 ab	17.7 ± 0.9 a	21.9 ± 0.8 b
*h* (°)	105.0 ± 0.5 b	104.9 ± 0.3 b	104.8 ± 0.3 b	104.6 ± 0.2 b	100.1 ± 0.5 a

Data are means ± standard error. Means followed by different letters across the treatments (within rows) are significantly different (*p* < 0.05). (g), Grams; ΔA, DA index; (°Bx), Brix; (N), Newton. L* (lightness), where values vary from 0 (black) to 100 (white); a*, (positive values represent red and negative green); b* (positive values represent yellow and negative values represent blue); C* (colorfulness), where higher values represent a more intense color and *h*° (hue angle), which is expressed in degrees. K1, water control; K2, juglone extraction medium and vehicle control (0.17% dimethyl sulfoxide (DMSO)), 0.17% ethanol in H_2_O; 10-4 positive control treatment with pure juglone prepared for final juglone concentration of 100 µM in the extraction medium; 10-3 positive control treatment with pure juglone prepared for final juglone concentration of 1 mM; LEAF 10-4 leaf juglone extract prepared for final juglone concentration of 100 µM in the extraction medium.

**Table 3:** Comparison of individual phenolic compounds and phenolic groups in fruit of *C. sativus* across different sampling days.

Phenolic Compound	Quantification According to Treatment (mg/kg Fresh Weight)										
	Day 0	Day 3					Day 6				
	SUM	K1	K2	LEAF 10-4	10-4	10-3	K1	K2	LEAF 10-4	10-4	10-3
Ferulic acid hexoside derivative	0.41 ± 0.01 a	0.67 ± 0.01 ac	0.47 ± 0.01 ab	1.07 ± 0.06 c	0.63 ± 0.03 ab	0.72 ± 0.05 ac	0.46 ± 0.02 ab	0.55 ± 0.01 ab	0.61 ± 0.01 ab	0.86 ± 0.04 bc	5.10 ± 0.26 d
Meloside A (Isovitexin-2-*O*-glucoside)	0.22 ± 0.06 a	0.46 ± 0.06 a	0.17 ± 0.11 a	0.61 ± 0.01 a	0.71 ± 0.04 a	1.04 ± 0.02 a	0.12 ± 0.06 a	0.13 ± 0.05 a	0.21 ± 0.05 a	0.36 ± 0.04 a	8.13 ± 0.70 b
Sinapic acid hexoside derivative	0.51 ± 0.01 ab	0.85 ± 0.02 d	0.50 ± 0.00 ab	0.60 ± 0.03 bc	0.63 ± 0.01 bc	0.72 ± 0.01 cd	0.42 ± 0.02 a	0.51 ± 0.02 ab	0.56 ± 0.01 b	0.50 ± 0.02 ab	1.80 ± 0.08 e
Feruoyl hexoside	0.02 ± 0.01 a	0.03 ± 0.00 a	0.01 ± 0.00 a	0.01 ± 0.00 a	0.01 ± 0.00 a	0.02 ± 0.01 a	0.01 ± 0.00 a	0.01 ± 0.00 a	0.01 ± 0.00 a	0.00 ± 0.00 a	0.45 ± 0.03 b
Saponarin (isovitexin-7-*O*-glucoside)-4-*O*-glucoside	0.07 ± 0.02 a	0.41 ± 0.10 c	0.14 ± 0.02 ab	0.14 ± 0.09 ab	0.15 ± 0.02 ab	0.28 ± 0.00 bc	0.09 ± 0.01 ab	0.12 ± 0.01 ab	0.17 ± 0.02 ab	0.13 ± 0.04 ab	1.40 ± 0.09 d
Isovitexin-2-*O*-(6-(E)-feruoyl)-dihexoside 1	0.53 ± 0.08 ab	0.71 ± 0.20 ab	0.53 ± 0.04 ab	0.84 ± 0.19 b	1.00 ± 0.02 b	0.46 ± 0.00 ab	0.31 ± 0.01 a	0.41 ± 0.09 a	0.57 ± 0.07 ab	0.52 ± 0.06 ab	7.82 ± 0.15 c
Isovitexin-2-*O*-(6-(E)-*p*-coumaroyl)-dihexoside 1	0.14 ± 0.0.03 ab	0.19 ± 0.10 ab	0.17 ± 0.01 ab	0.22 ± 0.10 ab	0.32 ± 0.02 b	0.13 ± 0.01 ab	0.01 ± 0.01 a	0.06 ± 0.04 ab	0.08 ± 0.03 ab	0.00 ± 0.00 a	1.10 ± 0.10 c
Isovitexin-2-*O*-(6-(E)-feruoyl)-dihexoside 2	4.23 ± 0.03 b	7.50 ± 0.13 e	4.37 ± 0.08 bc	5.33 ± 0.26 cd	5.56 ± 0.12 d	5.71 ± 0.10 d	2.60 ± 0.06 a	3.05 ± 0.06 a	2.67 ± 0.07 a	2.50 ± 0.12 a	22.16 ± 0.66 f
Isovitexin-2-*O*-(6-(E)-*p*-coumaroyl)-dihexoside 2	2.82 ± 0.02 b	5.00 ± 0.09 e	2.91 ± 0.05 bc	3.55 ± 0.17 cd	3.71 ± 0.08 d	3.81 ± 0.07 d	1.74 ± 0.04 a	2.03 ± 0.04 a	1.78 ± 0.05 a	1.67 ± 0.08 a	14.77 ± 0.43 f
Vicenin 2 (Isovitexin-8-C-glucoside)	0.48 ± 0.02 e	0.33 ± 0.01 bc	0.34 ± 0.02 bc	0.35 ± 0.02 bc	0.44 ± 0.03 de	0.27 ± 0.00 b	0.16 ± 0.01 a	0.16 ± 0.01 a	0.14 ± 0.01 a	0.16 ± 0.01 a	0.39 ± 0.02 cd
Isoscoparin-2-*O*-(6-(E)-feruoyl)-rhamnoside	0.54 ± 0.02 a	0.89 ± 0.04 a	0.50 ± 0.00 a	0.75 ± 0.04 a	0.90 ± 0.01 a	0.75 ± 0.01 a	0.35 ± 0.02 a	0.34 ± 0.00 a	0.31 ± 0.01 a	0.39 ± 0.01 a	8.36 ± 0.52 b
Isovitexin-2-*O*-(6-(E)-*p*-coumaroyl)-glucoside	1.91 ± 0.03 c	2.49 ± 0.16 d	1.61 ± 0.06 bc	1.92 ± 0.11 c	2.12 ± 0.05 cd	1.83 ± 0.03 c	1.00 ± 0.05 a	1.14 ± 0.03 ab	0.87 ± 0.03 a	1.04 ± 0.03 ab	13.79 ± 0.32 c
Isorhamnetin-3-*O*-glucoside	0.13 ± 0.01 a	0.90 ± 0.02 b	0.31 ± 0.06 ab	0.09 ± 0.05 a	0.32 ± 0.04 ab	0.53 ± 0.14 ab	0.35 ± 0.03 ab	0.42 ± 0.00 ab	0.22 ± 0.09 a	0.18 ± 0.13 a	7.47 ± 0.32 c
Total hydroxycinnamic acids	0.95 ± 0.01 a	1.56 ± 0.02 bc	0.98 ± 0.01 a	1.68 ± 0.09 c	1.27 ± 0.02 ac	1.45 ± 0.05 ac	0.89 ± 0.03 a	1.06 ± 0.03 ab	1.18 ± 0.01 ac	1.37 ± 0.05 ac	7.35 ± 0.36 d
Total flavones	10.94 ± 0.17 cd	17.99 ± 0.39 e	10.74 ± 0.12 bd	13.71 ± 0.85 de	14.90 ± 0.29 de	14.30 ± 0.23 de	6.39 ± 0.13 a	7.45 ± 0.25 abc	6.80 ± 0.19 ab	6.78 ± 0.34 ab	77.93 ± 2.69 f
Total flavonols	0.13 ± 0.01 a	0.90 ± 0.02 b	0.31 ± 0.06 ab	0.09 ± 0.05 a	0.32 ± 0.04 ab	0.53 ± 0.14 ab	0.35 ± 0.03 ab	0.42 ± 0.00 ab	0.22 ± 0.09 a	0.18 ± 0.13 a	7.47 ± 0.32 c
TAPC	12.02 ± 0.17 ab	20.44 ± 0.43 c	12.02 ± 0.18 ab	15.47 ± 0.96 bc	16.49 ± 0.30 bc	16.28 ± 0.27 bc	7.63 ± 0.14 a	8.93 ± 0.27 a	8.20 ± 0.27 a	8.33 ± 0.53 a	92.75 ± 3.34 d

Data are means ± standard error. Means followed by different letters across the treatments (within row) are significantly different (*p* < 0.05). TAPC, sum of the total analyzed phenolic content; total flavones, sum of the total flavones content; total flavonols, sum of the total flavonols content; total hydroxycinnamic acids, sum of the total hydroxycinnamic acids; SUM, fruit collected prior to adding any treatments from all plants; K1, water control; K2, juglone extraction medium and vehicle control (0.17% dimethyl sulfoxide (DMSO)), 0.17% ethanol in H_2_O; 10-4 positive control treatment with pure juglone prepared for final juglone concentration of 100 µM in the extraction medium; 10-3 positive control treatment with pure juglone prepared for final juglone concentration of 1 mM; LEAF 10-4 leaf juglone extract prepared for final juglone concentration of 100 µM in the extraction medium.

**Table 4:** Comparison of individual phenolic compounds and phenolic groups in leaves of *C. sativus* across different sampling days.

Phenolic Compound	Quantification According to Treatment (mg/kg Dry Weight)				
	day 0				
	K1	K2	LEAF 10-4	10-4	10-3
Caffeic acid derivative 1	4.2 ± 1.1 a	6.4 ± 2.0 ab	7.5 ± 1.0 ac	8.4 ± 1.4 ac	6.2 ± 1.9 a
Meloside A (Isovitexin-2-*O*-glucoside) 1	76.3 ± 17.8 ab	100.3 ± 23.9 ab	60.2 ± 11.8 a	76.7 ± 6.8 ab	110.0 ± 23.0 abc
Caffeic acid derivative 2	10.8 ± 4.4 abc	17.2 ± 5.2 ad	8.1 ± 2.3 ab	15.1 ± 1.5 ad	11.0 ± 2.5 abc
Ferulic acid hexoside derivative	13.2 ± 5.0 ab	15.1 ± 4.2 ab	12.0 ± 3.9 ab	18.0 ± 1.8 ab	14.7 ± 4.3 ab
Meloside A (Isovitexin-2-*O*-glucoside) 2	444.1 ± 61.8 bcd	473.9 ± 35.5 cde	331.9 ± 34.5 ac	334.3 ± 18.3 ac	570.9 ± 88.8 cde
Isoscoparin-2-*O*-(6-(E)-feruoyl)-glucoside	246.2 ± 33.4 cf	221.5 ± 14.7 ce	179.6 ± 17.6 acd	232.9 ± 11.3 cf	282.1 ± 43.3 def
Sinapic acid hexoside derivative	5.6 ± 2.7 a	10.0 ± 2.4 a	7.3 ± 2.2 a	12.2 ± 2.4 ab	7.5 ± 2.2 a
Saponarin (isovitexin-7-O-glucoside)-4-*O*-glucoside	75.8 ± 20.4 ac	113.2 ± 11.9 bcd	80.1 ± 13.1 ac	120.1 ± 11.8 bcd	117.8 ± 22.5 bcd
Isoscoparin-2-*O*-(6-(E)-feruoyl)-dihexoside 1	294.7 ± 57.9 bc	389.5 ± 5.5 cde	279.4 ± 27.3 bc	329.5 ± 13.5 bd	400.2 ± 56.8 cde
Isovitexin-2-*O*-(6-(E)-feruoyl)-dihexoside 1	354.1 ± 34.7 bc	467.5 ± 5.7 cd	338.1 ± 12.8 bc	475.4 ± 17.0 cd	362.0 ± 52.6 bc
Isovitexin-2-*O*-(6-(E)-*p*-coumaroyl)-dihexoside 1	339.0 ± 17.2 bc	435.4 ± 10.2 bde	359.8 ± 24.3 bc	450.5 ± 26.8 bde	392.0 ± 54.6 bc
Isoscoparin-2-*O*-(6-(E)-feruoyl)-dihexoside 2	302.6 ± 34.4 bd	337.1 ± 22.2 cd	237.3 ± 15.2 bc	316.7 ± 17.0 cd	303.6 ± 44.1 bd
Isovitexin-2-*O*-(6-(E)-feruoyl)-dihexoside 2	1088.0 ± 185.3 c	1229.0 ± 97.6 cd	865.9 ± 86.4 bc	1027.7 ± 19.2 bc	872.7 ± 85.2 bc
Isovitexin-2-*O*-(6-(E)-*p*-coumaroyl)-dihexoside 2	615.5 ± 34.3 ac	769.9 ± 14.9 bcd	547.2 ± 26.9 ab	734.7 ± 33.3 bcd	764.3 ± 129.0 bcd
Isoscoparin-2-*O*-(6-(E)-feruoyl)-dihexoside 3	37.7 ± 13.0 ad	62.4 ± 11.2 cd	55.3 ± 7.9 bcd	80.1 ± 15.0 d	44.4 ± 10.5 bcd
Isovitexin-2-*O*-(6-(E)-feruoyl)-dihexoside 3	75.4 ± 20.2 bc	84.4 ± 13.2 bcd	75.6 ± 10.2 bc	94.2 ± 15.3 cd	89.6 ± 19.3 bcd
Isovitexin-2-*O*-(6-(E)-*p*-coumaroyl)-dihexoside 3	85.6 ± 19.4 bd	103.0 ± 14.1 be	81.7 ± 8.4 bc	93.3 ± 13.3 be	99.1 ± 18.9 be
Isoscoparin-2-*O*-(6-(E)-feruoyl)-rhamnoside-glucoside	88.0 ± 26.8 bcd	98.3 ± 19.0 cd	89.5 ± 9.9 bcd	109.1 ± 16.1 cd	103.0 ± 19.7 cd
Meloside A (Isovitexin-2-*O*-glucoside)-glucoside	74.6 ± 17.3 bcd	63.9 ± 9.2 bcd	74.0 ± 6.5 bcd	84.3 ± 9.0 d	80.1 ± 17.7 cd
Meloside A (Isovitexin-2-*O*-glucoside)-glucoside derivative	36.2 ± 19.4 acd	88.5 ± 19.8 cf	59.7 ± 10.1 acef	75.4 ± 19.1 bcef	63.7 ± 19.4 acef
Isovitexin-8-C-galactoside	28.9 ± 20.6 ab	33.8 ± 14.6 ab	29.4 ± 5.9 ab	32.1 ± 16.5 ab	24.7 ± 9.1 ab
Vicenin 2 (Isovitexin-8-C-glucoside)	533.8 ± 17.3 cde	687.3 ± 25.8 efg	864.8 ± 38.7 g	1169.7 ± 44.3 h	645.9 ± 85.5 df
Isoscoparin-2-*O*-glucoside	74.2 ± 20.0 dg	70.2 ± 15.3 def	105.0 ± 4.8 fg	125.7 ± 15.2 g	73.6 ± 15.1 dg
*p*-Coumaric acid	75.6 ± 3.3 bd	85.1 ± 2.5 cd	124.1 ± 4.4 g	120.7 ± 4.3 fg	95.0 ± 11.5 cde
Isovitexin-2-*O*-(6-(E)-feruoyl)-hexoside derivative	58.0 ± 12.6 ac	89.0 ± 16.0 cd	77.7 ± 7.1 bcd	87.7 ± 9.6 cd	60.8 ± 18.3 ac
Isoscoparin-2-*O*-(6-(E)-feruoyl)-derivative	98.5 ± 8.6 ce	81.3 ± 9.4 bcd	100.7 ± 5.2 ce	109.0 ± 9.1 ce	98.5 ± 22.7 ce
Isoscoparin-2-*O*-(6-(E)-feruoyl)-rhamnoside	150.0 ± 11.2 cdf	102.6 ± 10.5 bc	158.4 ± 8.5 cdf	180.7 ± 9.6 ef	151.7 ± 28.6 cdf
Isovitexin-2-*O*-(6-(E)-feruoyl)-glucoside	257.5 ± 2.6 bc	296.8 ± 5.5 bde	269.2 ± 10.6 bd	338.8 ± 12.2 bf	275.7 ± 36.9 bd
Isovitexin-2-*O*-(6-(E)-*p*-coumaroyl)-glucoside	466.8 ± 0.6 bc	471.5 ± 9.8 bc	525.3 ± 18.5 bc	739.1 ± 19.4 e	436.2 ± 56.8 bc
Total flavones	5901.5 ± 485.6 bc	6869.6 ± 377.2 bde	5846.0 ± 352.9 bc	7417.8 ± 372.3 bde	6422.7 ± 968.9 bd
Total hydroxycinnamic acids	109.3 ± 15.5 ad	133.8 ± 14.1 bde	158.9 ± 13.4 bde	174.4 ± 11.3 cde	134.3 ± 20.5 bde
TAPC	6010.8 ± 501.1 c	7003.4 ± 387.3 cde	6004.9 ± 365.7 c	7592.2 ± 383.5 cde	6557.0 ± 988.1 cd
	day 3				
	K1	K2	LEAF 10-4	10-4	10-3
Caffeic acid derivative 1	16.3 ± 1.7 ac	30.6 ± 1.8 c	23.1 ± 3.7 ac	9.9 ± 0.6 ac	10.9 ± 0.6 ac
Meloside A (Isovitexin-2-*O*-glucoside) 1	131.4 ± 3.3 abd	241.5 ± 32.8 e	167.1 ± 12.4 be	78.1 ± 1.7 ab	51.0 ± 6.4 a
Caffeic acid derivative 2	17.3 ± 1.4 ad	26.4 ± 2.1 d	14.8 ± 2.8 ad	8.6 ± 0.1 ab	5.3 ± 0.2 a
Ferulic acid hexoside derivative	23.1 ± 4.8 ab	26.3 ± 1.9 ab	19.0 ± 5.2 ab	11.7 ± 0.3 ab	13.0 ± 0.6 a
Meloside A (Isovitexin-2-*O*-glucoside) 2	587.4 ± 46.5 cde	968.7 ± 87.8 fg	729.1 ± 17.3 ef	367.3 ± 9.5 bc	81.7 ± 5.8 a
Isoscoparin-2-*O*-(6-(E)-feruoyl)-glucoside	289.6 ± 16.6 ef	271.9 ± 23.8 def	242.1 ± 7.4 cf	156.7 ± 2.2 ac	76.9 ± 5.8 a
Sinapic acid hexoside derivative	14.4 ± 1.0 abc	15.2 ± 2.0 abc	11.1 ± 0.8 ab	13.3 ± 0.5 abc	12.8 ± 0.8 ab
Saponarin (isovitexin-7-*O*-glucoside)-4-*O*-glucoside	144.3 ± 10.5 ce	159.3 ± 13.8 de	113.4 ± 11.6 bcd	68.3 ± 1.2 ab	38.3 ± 12.7 a
Isoscoparin-2-*O*-(6-(E)-feruoyl)-dihexoside 1	352.7 ± 64.6 bd	551.9 ± 43.1 ef	462.0 ± 4.5 cde	322.8 ± 6.3 bd	75.9 ± 3.3 a
Isovitexin-2-*O*-(6-(E)-feruoyl)-dihexoside 1	440.7 ± 15.6 bd	653.2 ± 54.8 ef	430.3 ± 9.7 bd	408.1 ± 10.9 bd	126.0 ± 7.3 a
Isovitexin-2-*O*-(6-(E)-*p*-coumaroyl)-dihexoside 1	420.0 ± 43.1 bd	620.9 ± 52.7 ef	496.7 ± 12.6 cde	402.0 ± 15.8 bd	123.8 ± 5.9 a
Isoscoparin-2-*O*-(6-(E)-feruoyl)-dihexoside 2	362.8 ± 22.9 cde	325.1 ± 22.6 cd	301.4 ± 6.6 bd	275.2 ± 4.9 bd	104.6 ± 4.4 a
Isovitexin-2-*O*-(6-(E)-feruoyl)-dihexoside 2	1032.0 ± 143.7 bc	1138.5 ± 84.1 cd	1027.5 ± 98.5 bc	1007.1 ± 51.8 bc	329.9 ± 3.1 a
Isovitexin-2-*O*-(6-(E)-*p*-coumaroyl)-dihexoside 2	796.5 ± 67.4 bcd	1419.5 ± 153.5 e	905.2 ± 140.4 bcd	688.4 ± 62.3 bc	187.7 ± 22.0 a
Isoscoparin-2-*O*-(6-(E)-feruoyl)-dihexoside 3	62.1 ± 4.4 cd	50.7 ± 6.4 bcd	40.4 ± 6.7 ad	30.0 ± 2.5 ac	13.1 ± 0.8 a
Isovitexin-2-*O*-(6-(E)-feruoyl)-dihexoside 3	113.3 ± 8.6 cd	109.4 ± 12.3 cd	93.3 ± 8.2 cd	68.6 ± 3.7 ac	16.7 ± 1.0 a
Isovitexin-2-*O*-(6-(E)-*p*-coumaroyl)-dihexoside 3	116.3 ± 7.7 cde	149.8 ± 13.2 ef	123.4 ± 6.9 cde	96.2 ± 4.3 be	21.6 ± 1.1 a
Isoscoparin-2-*O*-(6-(E)-feruoyl)-rhamnoside-glucoside	124.7 ± 11.7 cd	109.7 ± 12.6 cd	91.6 ± 9.0 bcd	74.2 ± 3.9 ad	17.2 ± 2.0 a
Meloside A (Isovitexin-2-*O*-glucoside)-glucoside	89.1 ± 8.7 d	70.7 ± 7.7 bcd	81.7 ± 2.8 cd	53.0 ± 4.2 ad	16.9 ± 0.5 a
Meloside A (Isovitexin-2-*O*-glucoside)-glucoside derivative	83.5 ± 8.6 cf	122.3 ± 16.0 f	50.7 ± 8.6 ace	52.7 ± 6.7 ace	7.4 ± 1.3 a
Isovitexin-8-C-galactoside	48.0 ± 5.3 ab	67.7 ± 9.4 b	26.8 ± 7.3 ab	39.5 ± 4.4 ab	21.2 ± 0.6 ab
Vicenin 2 (Isovitexin-8-C-glucoside)	508.2 ± 22.5 cde	752.8 ± 59.3 fg	469.2 ± 11.3 bd	424.7 ± 16.1 bc	34.3 ± 0.2 a
Isoscoparin-2-*O*-glucoside	99.3 ± 14.2 eg	59.4 ± 10.4 cdef	57.1 ± 5.4 bdef	34.6 ± 4.3 ad	1.8 ± 0.2 a
*p*-Coumaric acid	83.5 ± 2.5 cd	97.7 ± 5.8 def	111.6 ± 0.5 eg	54.2 ± 3.0 b	9.0 ± 0.6 a
Isovitexin-2-*O*-(6-(E)-feruoyl)-hexoside derivative	71.8 ± 9.2 bcd	202.2 ± 23.0 e	62.5 ± 2.0 ac	76.7 ± 3.4 bcd	9.2 ± 3.0 a
Isoscoparin-2-*O*-(6-(E)-feruoyl)-derivative	124.1 ± 9.8 de	75.5 ± 5.7 ac	101.6 ± 0.4 ce	76.2 ± 3.0 ac	30.5 ± 3.4 a
Isoscoparin-2-*O*-(6-(E)-feruoyl)-rhamnoside	166.5 ± 9.7 df	133.7 ± 9.4 cde	192.0 ± 3.0 f	114.1 ± 2.8 cd	39.1 ± 2.9 a
Isovitexin-2-*O*-(6-(E)-feruoyl)-glucoside	255.6 ± 11.8 b	352.2 ± 20.2 bf	363.4 ± 6.1 cdf	283.5 ± 6.1 bde	56.3 ± 4.3 a
Isovitexin-2-*O*-(6-(E)-*p*-coumaroyl)-glucoside	406.0 ± 15.9 b	692.6 ± 40.5 de	555.7 ± 8.1 cd	447.5 ± 6.2 bc	68.6 ± 6.0 a
Total flavones	6826.0 ± 364.8 bde	9299.3 ± 745.4 ef	7184.3 ± 137.0 bde	5645.5 ± 112.4 b	1537.9 ± 91.9 a
Total hydroxycinnamic acids	154.7 ± 8.8 bde	196.2 ± 12.1 e	179.7 ± 12.2 de	97.7 ± 2.5 abc	51.0 ± 2.1 a
TAPC	6980.6 ± 373.4 cde	9495.5 ± 756.5 ef	7364.0 ± 146.4 cde	5743.1 ± 114.5 bc	1588.9 ± 94.0 a
	day 6				
	K1	K2	LEAF 10-4	10-4	10-3
Caffeic acid derivative 1	29.9 ± 2.1 bc	145.6 ± 1.3 e	152.7 ± 8.7 e	80.0 ± 14.0 d	24.5 ± 0.6 ac
Meloside A (Isovitexin-2-*O*-glucoside) 1	256.1 ± 5.8 e	212.1 ± 14.1 de	383.0 ± 29.2 f	197.6 ± 32.7 cde	90.1 ± 6.4 ab
Caffeic acid derivative 2	22.7 ± 0.5 cd	26.6 ± 2.1 d	40.8 ± 2.0 e	21.5 ± 4.0 bd	10.9 ± 1.2 abc
Ferulic acid hexoside derivative	30.6 ± 1.4 ab	30.3 ± 3.9 ab	54.7 ± 6.6 c	33.0 ± 7.1 b	16.2 ± 1.4 ab
Meloside A (Isovitexin-2-*O*-glucoside) 2	1136.6 ± 42.1 gh	910.4 ± 25.7 fg	1334.1 ± 66.5 h	718.9 ± 106.2 df	172.3 ± 18.1 ab
Isoscoparin-2-*O*-(6-(E)-feruoyl)-glucoside	299.4 ± 2.4 ef	228.7 ± 13.0 cf	335.7 ± 15.2 f	212.0 ± 35.9 ac	107.3 ± 11.8 a
Sinapic acid hexoside derivative	24.5 ± 1.2 cd	14.4 ± 3.2 abc	27.6 ± 2.0 d	22.3 ± 4.5 bd	26.8 ± 1.3 d
Saponarin (isovitexin-7-*O*-glucoside)-4-*O*-glucoside	129.3 ± 6.7 bce	102.9 ± 13.1 acd	199.3 ± 12.6 e	100.4 ± 21.8 acd	37.8 ± 3.9 a
Isoscoparin-2-*O*-(6-(E)-feruoyl)-dihexoside 1	564.4 ± 19.0 ef	483.6 ± 11.9 df	650.2 ± 11.9 f	434.6 ± 64.3 cde	169.5 ± 12.5 ab
Isovitexin-2-*O*-(6-(E)-feruoyl)-dihexoside 1	576.7 ± 15.1 df	553.2 ± 12.8 de	746.2 ± 30.2 f	538.6 ± 84.2 de	275.4 ± 19.8 ab
Isovitexin-2-*O*-(6-(E)-*p*-coumaroyl)-dihexoside 1	591.8 ± 14.3 df	521.0 ± 17.9 cde	775.7 ± 26.3 f	515.5 ± 88.8 cde	261.7 ± 32.7 ab
Isoscoparin-2-*O*-(6-(E)-feruoyl)-dihexoside 2	380.4 ± 13.1 de	289.5 ± 9.5 bd	477.1 ± 16.9 e	357.6 ± 55.5 cde	187.8 ± 15.0 ab
Isovitexin-2-*O*-(6-(E)-feruoyl)-dihexoside 2	1371.7 ± 46.5 cd	1292.1 ± 41.2 cd	1646.5 ± 16.0 d	1307.4 ± 194.6 cd	556.0 ± 48.8 ab
Isovitexin-2-*O*-(6-(E)-*p*-coumaroyl)-dihexoside 2	1133.5 ± 49.1 de	1053.2 ± 10.4 ce	1428.4 ± 121.0 e	817.6 ± 132.0 bcd	479.9 ± 24.0 ab
Isoscoparin-2-*O*-(6-(E)-feruoyl)-dihexoside 3	40.7 ± 4.9 ad	30.0 ± 7.9 ac	58.3 ± 4.6 bcd	27.5 ± 5.0 ac	18.8 ± 9.3 ab
Isovitexin-2-*O*-(6-(E)-feruoyl)-dihexoside 3	92.2 ± 5.5 cd	78.3 ± 9.3 bc	140.3 ± 7.4 d	57.1 ± 8.4 ac	32.4 ± 6.8 ab
Isovitexin-2-*O*-(6-(E)-*p*-coumaroyl)-dihexoside 3	141.0 ± 3.9 de	141.3 ± 7.7 de	207.5 ± 8.2 f	111.8 ± 16.8 cde	47.7 ± 4.9 ab
Isoscoparin-2-*O*-(6-(E)-feruoyl)-rhamnoside-glucoside	94.7 ± 7.3 bcd	63.9 ± 7.0 ac	131.4 ± 7.4 d	62.6 ± 9.2 ac	29.7 ± 1.8 ab
Meloside A (Isovitexin-2-*O*-glucoside)-glucoside	61.8 ± 3.7 ad	45.7 ± 6.6 ad	84.2 ± 4.4 d	32.3 ± 4.3 ab	38.2 ± 9.4 ac
Meloside A (Isovitexin-2-*O*-glucoside)-glucoside derivative	85.7 ± 9.2 cf	96.2 ± 10.9 def	102.5 ± 7.7 ef	29.5 ± 1.6 ac	13.4 ± 3.4 ab
Isovitexin-8-C-galactoside	15.3 ± 7.7 a	30.5 ± 4.3 ab	45.4 ± 5.0 ab	6.8 ± 1.7 a	32.0 ± 3.0 ab
Vicenin 2 (Isovitexin-8-C-glucoside)	597.6 ± 5.8 cdf	779.2 ± 12.4 fg	663.5 ± 25.7 ef	318.0 ± 42.9 b	58.3 ± 2.5 a
Isoscoparin-2-*O*-glucoside	49.2 ± 7.5 ade	54.6 ± 7.4 adef	69.3 ± 5.0 def	12.6 ± 4.8 abc	3.8 ± 1.4 ab
*p*-Coumaric acid	70.8 ± 3.0 bc	98.9 ± 3.1 def	81.7 ± 2.4 cd	53.7 ± 8.4 b	15.1 ± 0.6 a
Isovitexin-2-*O*-(6-(E)-feruoyl)-hexoside derivative	76.3 ± 1.3 bcd	121.0 ± 5.4 d	95.7 ± 7.2 cd	28.3 ± 6.8 ab	13.6 ± 4.1 a
Isoscoparin-2-*O*-(6-(E)-feruoyl)-derivative	98.7 ± 2.9 ce	72.0 ± 9.0 ac	129.8 ± 7.5 e	65.4 ± 8.3 ac	46.7 ± 6.4 ab
Isoscoparin-2-*O*-(6-(E)-feruoyl)-rhamnoside	137.7 ± 1.9 cdf	132.1 ± 3.8 cde	176.0 ± 4.4 ef	110.6 ± 18.4 bd	54.7 ± 5.4 ab
Isovitexin-2-*O*-(6-(E)-feruoyl)-glucoside	370.1 ± 7.1 df	382.6 ± 4.7 ef	542.9 ± 9.5 g	407.6 ± 61.6 f	148.0 ± 7.4 a
Isovitexin-2-*O*-(6-(E)-*p*-coumaroyl)-glucoside	533.7 ± 9.2 bc	729.9 ± 3.5 e	889.9 ± 19.9 f	469.5 ± 71.4 bc	148.1 ± 8.3 a
Total flavones	8834.5 ± 159.7 df	8403.7 ± 235.8 cde	11,312.8 ± 385.9 f	6939.9 ± 1058.2 bde	3023.0 ± 232.4 a
Total hydroxycinnamic acids	178.5 ± 5.1 de	315.8 ± 11.4 f	357.6 ± 20.7 f	210.5 ± 36.5 e	93.5 ± 3.8 ab
TAPC	9012.9 ± 155.0 df	8719.6 ± 245.7 de	11,670.4 ± 394.3 f	7150.3 ± 1093.6 cde	3116.5 ± 236.2 ab

Data are means ± standard error. Means followed by different letters across the treatments (within individual compounds) are significantly different (*p* < 0.05). TAPC, sum of the total analyzed phenolic content; total flavones, sum of the total flavones content; total hydroxycinnamic acids, sum of the total hydroxycinnamic acids; K1, water control; K2, juglone extraction medium and vehicle control (0.17% dimethyl sulfoxide (DMSO)), 0.17% ethanol in H_2_O; 10-4 positive control treatment with pure juglone prepared for final juglone concentration of 100 µM in the extraction medium; 10-3 positive control treatment with pure juglone prepared for final juglone concentration of 1 mM; LEAF 10-4 leaf juglone extract prepared for final juglone concentration of 100 µM in the extraction medium.

**Table 5:** Comparison of individual phenolic compounds and phenolic groups in roots of *C. sativus* on the last sampling date.

Phenolic Compound	Quantification According to Treatment (mg/kg Dry Weight)				
	Day 6				
	K1	K2	LEAF 10-4	10-4	10-3
Benzoic acid	435.8 ± 8.1 e	125.3 ± 3.7 d	39.6 ± 1.1 c	28.0 ± 0.8 b	12.7 ± 0.6 a
Quercetin-3-*O*-rutinoside	26.4 ± 6.6 b	27.2 ± 6.8 b	2.4 ± 1.2 a	9.6 ± 1.6 ab	5.3 ± 0.6 a
Quercetin-3-*O*-rhamnoside	8.3 ± 0.9 a	40.8 ± 0.3 e	37.3 ± 0.2 d	13.4 ± 0.4 b	25.4 ± 1.4 c
Hydrogalic acid	18.8 ± 0.7 b	34.3 ± 0.3 c	98.0 ± 0.5 e	10.6 ± 0.5 d	41.1 ± 2.0 a
Juglone (5-hydroxy-1,4-naphthalenedione)	nd	nd	26.4 ± 1.3 a	22.8 ± 0.7 a	659.9 ± 18.1 b
Total hydroxybenzoic acids	454.6 ± 7.9 d	159.6 ± 3.9 c	137.6 ± 1.5 b	38.6 ± 1.1 a	53.7 ± 1.8 a
Total flavonols	34.7 ± 7.3 a	68.0 ± 7.0 b	39.6 ± 1.2 a	23.0 ± 1.5 a	30.7 ± 1.6 a
TAPCWJ	489.3 ± 14.0 c	227.6 ± 10.7 b	177.2 ± 2.2 b	84.4 ± 3.0 a	744.3 ± 18.5 d
TAPC	489.3 ± 14.0 d	227.6 ± 10.7 c	150.8 ± 2.1 b	61.6 ± 2.7 a	84.4 ± 2.4 a

Data are means ± standard error. Means followed by different letters across the treatments (within individual compounds) are significantly different (*p* < 0.05). TAPC, sum of the total analyzed phenolic content without juglone content; TAPCWJ, sum of the total analyzed phenolic content with juglone content; total flavones, sum of the total flavones content; total hydroxycinnamic acids, sum of the total hydroxycinnamic acids; K1, water control; K2, juglone extraction medium and vehicle control (0.17% dimethyl sulfoxide (DMSO)), 0.17% ethanol in H_2_O; 10-4 positive control treatment with pure juglone prepared for final juglone concentration of 100 µM in the extraction medium; 10-3 positive control treatment with pure juglone prepared for final juglone concentration of 1 mM; LEAF 10-4 leaf juglone extract prepared for final juglone concentration of 100 µM in the extraction medium.

## Data Availability

The data are available from the corresponding author.

## Supplementary Materials

The following are available online at https://www.mdpi.com/article/10.3390/foods12020371/s1. Figure S1: Chromatogram of the compounds identified in the fruit of *C. sativus* at 280 nm, Figure S2: Chromatogram of the compounds identified in the leaves of *C. sativus* at 280 nm, and Figure S3: Chromatogram of the compounds identified in the roots of *C. sativus* at 280 nm.
