# Supplementary material for: Effect of Juglone and Other Allelochemicals in Walnut Leaves on Yield, Quality and Metabolites of Snack Cucumber (Cucumis sativus L.)
Source: Foods. 2023 Jan 12;12(2):371. doi: 10.3390/foods12020371 (PMC9858246; doi:10.3390/foods12020371)
Supplement: Supplementary file 1 [file foods-12-00371-s001.zip › foods-2108413-supplementary.pdf]

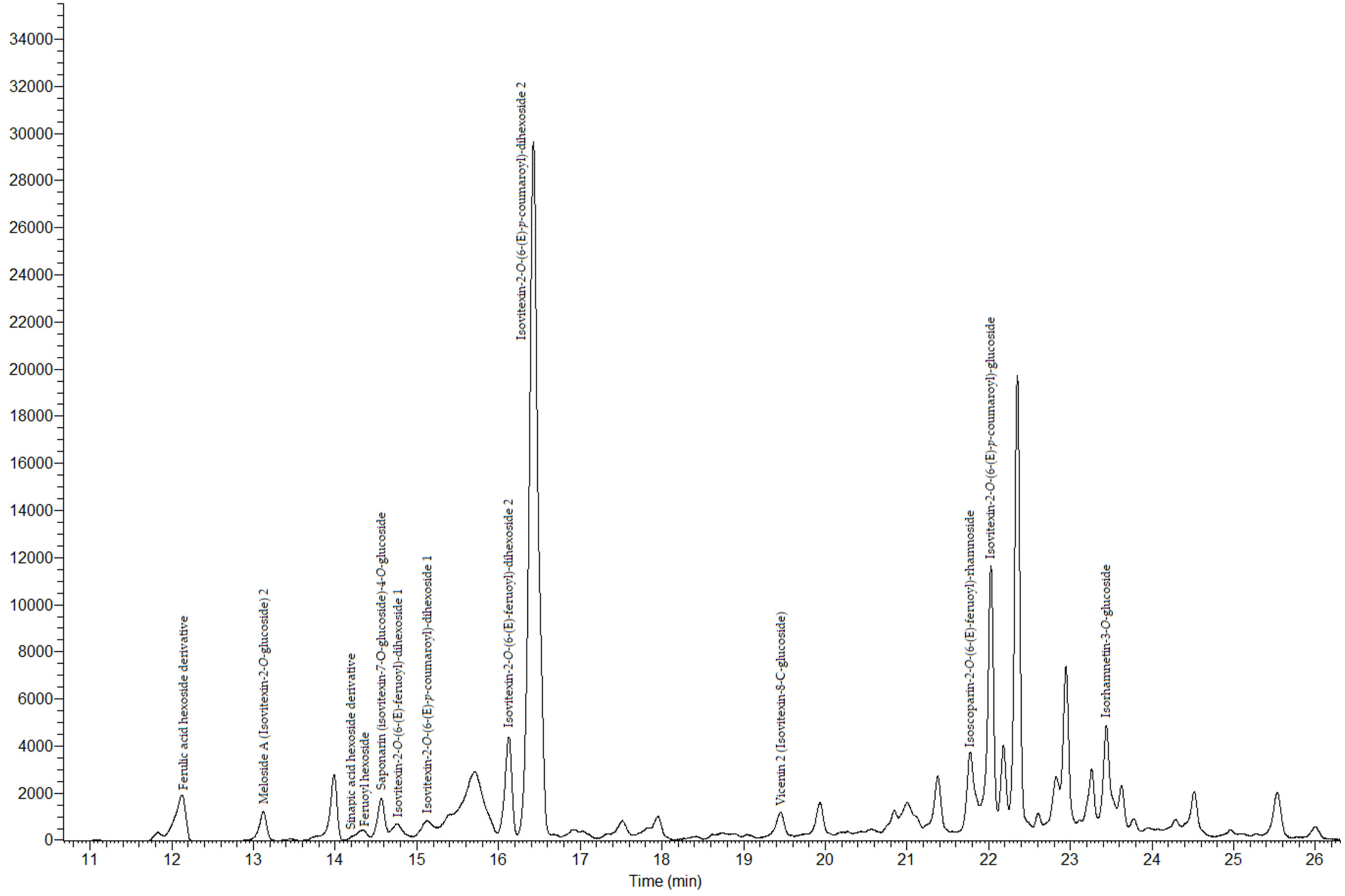

Figure S1. Chromatogram of the compounds identified in the fruit of *C. sativus* at 280 nm.

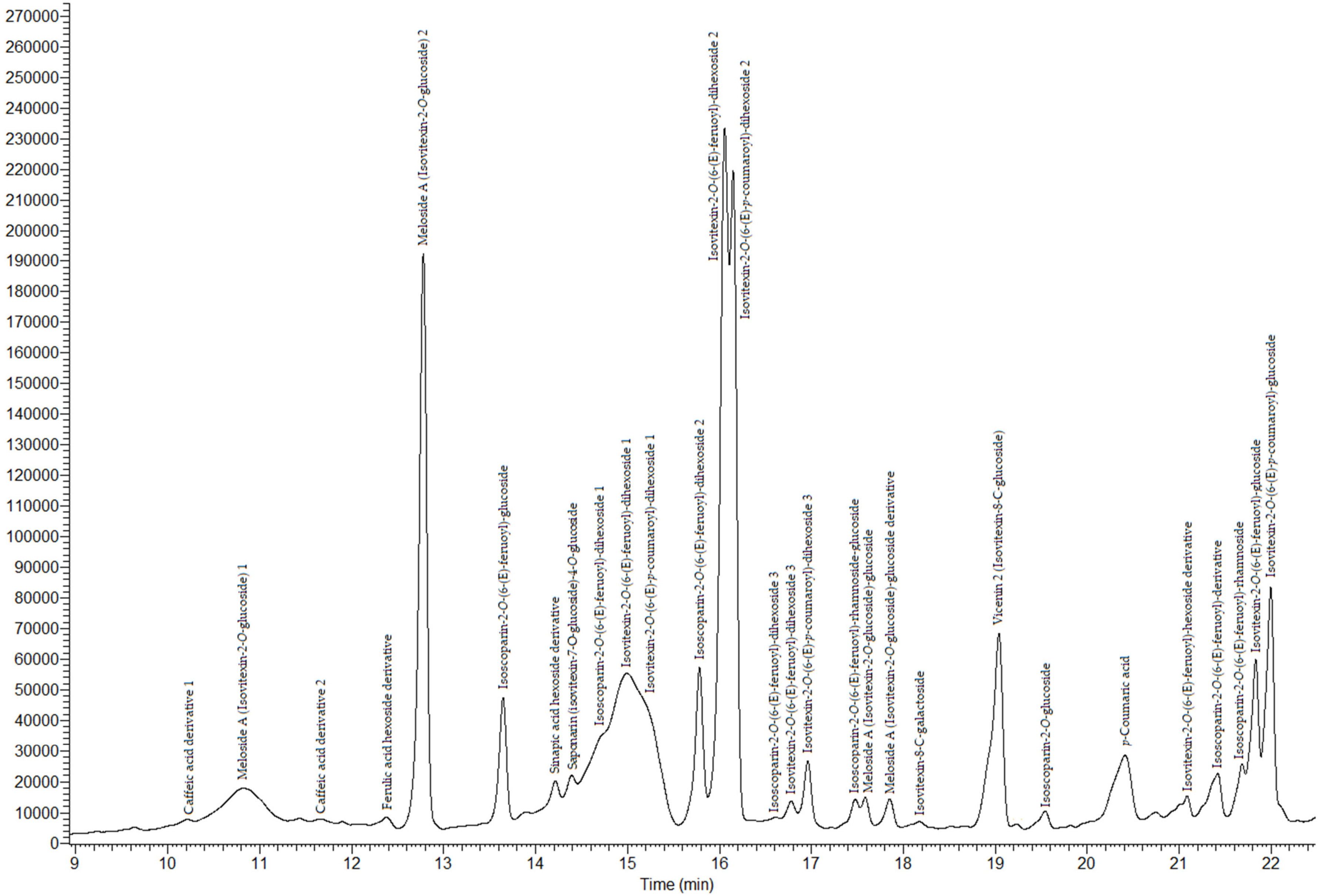

Figure S2. Chromatogram of the compounds identified in the leaves of *C. sativus* at 280 nm.

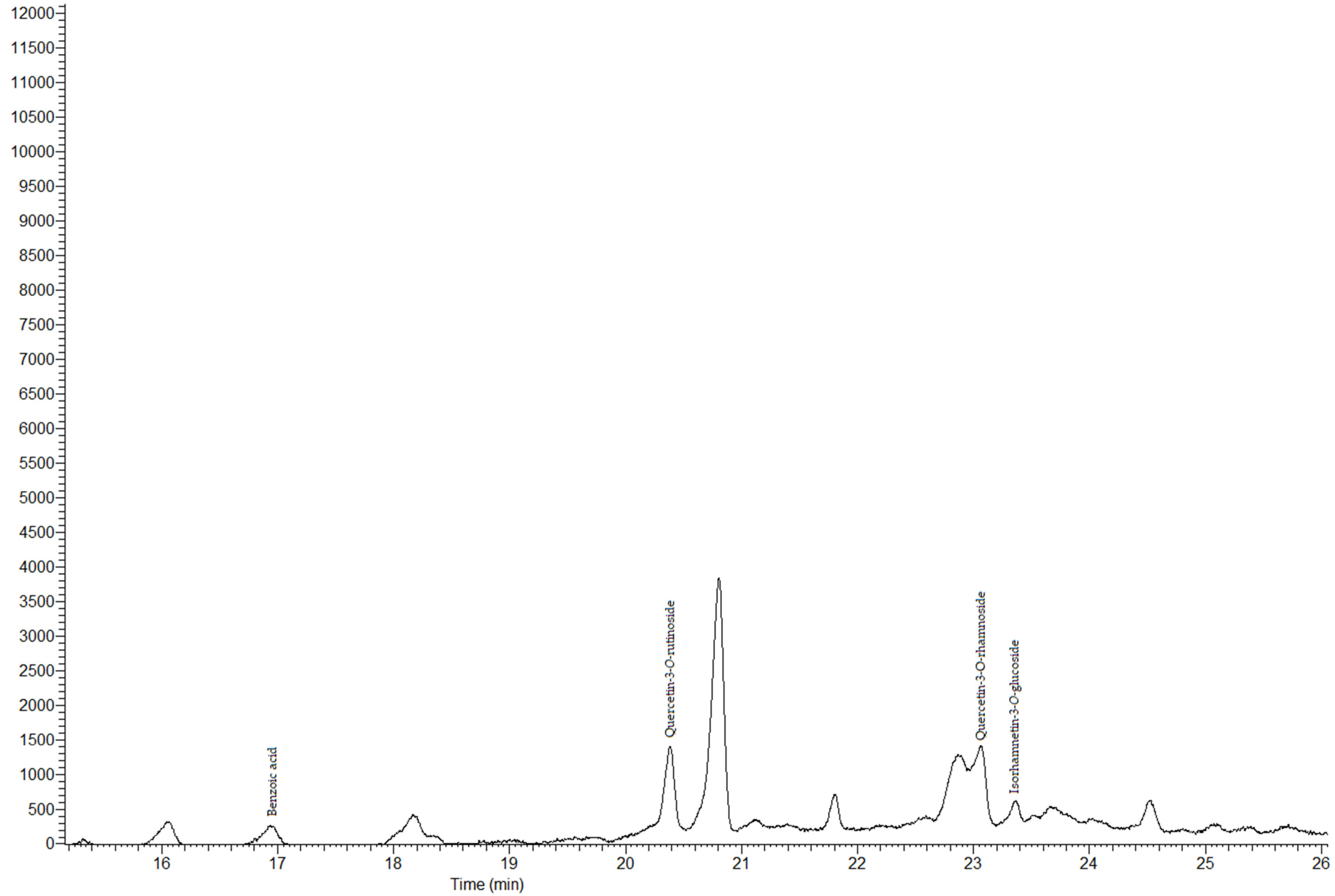

Figure S3. Chromatogram of the compounds identified in the roots of *C. sativus* at 280 nm.
